# Contrasting Sonodegradation and Anodic Oxidation of Sulfonamides in Water: Degradation Routes, Matrix Effects, and Theoretical Study

**DOI:** 10.3390/molecules31081292

**Published:** 2026-04-15

**Authors:** Efraím A. Serna-Galvis, Ricardo A. Torres-Palma

**Affiliations:** 1 Grupo de Investigación en Remediación Ambiental y Biocatálisis (GIRAB), Instituto de Química, Facultad de Ciencias Exactas y Naturales, Universidad de Antioquia UdeA, Calle 70 No. 52-21, Medellín 050010, Colombia; 2 Departamento de Química, Facultad de Ciencias Naturales y Exactas, Universidad del Valle, Calle 13 No. 100-00, Santiago de Cali 760032, Colombia

**Keywords:** antibiotics degradation, ACC, electrochemistry, sonochemistry, photochemistry, sulfonamides, water treatment

## Abstract

Mid-high-frequency ultrasound (375 kHz) and anodic oxidation at low current intensity (<50 mA, NaCl as the supporting electrolyte) were employed to treat sulfonamide antibiotics (sulfamethoxazole—SMX and sulfacetamide—SAM). The sonodegradation involved HO**•**, while electrogenerated HClO was mainly responsible for the antibiotics’ elimination in the electrochemical process. A comparison of the processes evidenced that the degradation of SMX by ultrasound was faster due to its higher hydrophobicity. In contrast, in the electrochemical system, the SAM degradation was more efficient, which was associated with a higher reactivity of its acetamide moiety toward HClO. Interestingly, SMX was selectively sonodegraded in synthetic hospital wastewater and seawater, whereas the matrix components strongly accelerated the electrochemical degradation but affected the process performance in the hospital wastewater. On the other hand, theoretical analyses of atomic charge indicated that the central S-N bond, the N and aromatic ring in the aniline moiety, the C=C bond, and methyl groups in the isoxazole groups on SMX are the most susceptible moieties to the attacks by HO**•** and HClO. Furthermore, for the typical byproducts, calculations of the probability of being active against bacteria were slightly lower than that of the parent pharmaceutical, even being much lower for the byproducts from the electrochemical treatment.

## 1. Introduction

The high consumption and inability (or absence) of municipal treatment plants to completely remove active pharmaceutical ingredients (e.g., antibiotics) from wastewater have led to the omnipresence of those substances in the natural water [1,2,3,4]. Antibiotics in the environment are a problem of increasing importance due to the induction of toxic effects on aquatic organisms and promotion of the proliferation of antimicrobial resistance [4,5]. Among highly consumed antibiotics worldwide, we can find the sulfonamides therapeutic class (which includes SMX and SAM). This antibiotic class is utilized for healing illnesses in humans caused by several bacterial infections, and after consumption, a fraction of the active principle is excreted via urine [6]; for example, between 15 and 30% of ingested SMX is excreted [7]. Thus, hospital wastewater is a relevant source of sulfonamide discharge, which ends up in municipal sewage systems or is directly discharged into environmental water. For instance, SMX is frequently detected in hospital wastewater [7,8,9], effluents of sewage treatment plants, and even in environmental water [7]. Some works report the presence of SMX in effluents of hospitals in Colombia in the range of 0.09–198.53 µg L^−1^ [8]. Moreover, SMX is one of the antibiotics with the lowest removal efficiency in wastewater treatment plants, and consequently, this sulfonamide has been measured in sewage treatment plant effluents at levels of 0.047–309 µg L^−1^ in South Korea, 106–405 ng L^−1^ in Guangdong in China [10], 350–650 ng L^−1^ in Medellín and Bogotá in Colombia [9], 76–100 ng L^−1^ in South Cyprus, 118.5 ng L^−1^ in Dresden in Germany, 80 ng L^−1^ in Volos in Greece, 340–1679 76–100 ng L^−1^ in Coimbra in Portugal, and 120–420 ng L^−1^ in Udupi in India [7].

Despite the aerobic or anaerobic biological processes (which are the typical secondary systems in municipal treatment plants) being good systems to transform biodegradable organic matter such as proteins, carbohydrates, or lipids into energy resources (e.g., biogas, [11]), these processes are not fully efficient to completely remove sulfonamide antibiotics [7]. Then, this class of antibiotics reaches environmental water. Indeed, high concentrations of SMX have been observed in the Hai River system, China (up to 4.87 µg L^−1^), and in the Kshipra River, India (up to 4.66 µg L^−1^). Also, for surface water, SMX was found in high levels of 2.42 µg L^−1^ and 3.066 µg L^−1^ in the João Mendes River, Brazil, and the Charmoise River, France, respectively. Moreover, a concentration of 1.285 µg L^−1^ for SMX in Yaoundé, Cameroon, in groundwater samples has been reported. Furthermore, several studies of sulfonamides in surface water and groundwater reveal that SMX exhibits the highest concentrations, reaching levels up to 142.6 µg L^−1^ [12]. In the case of SAM, this sulfonamide has been determined in surface watersheds in China (0.13–0.81 ng L^−1^) and in Lake Victoria in Uganda (1–13 ng L^−1^) [13].

In addition to their utilization in human health, sulfonamides are frequently used in aquaculture to prevent and treat diseases [10]. In fact, seawater aquaculture (also known as mariculture, for farming of marine organisms like fish, shellfish, and seaweed in saltwater; and which also uses coastal ponds, suspended ropes, or offshore net pens to produce seafood) is another source of environmental discharge of sulfonamides [7]. Thus, concentrations of SMX between 1.5 and 3.1 µg L^−1^ have been determined in seawater matrices in France, Brazil, and Spain [10].

It must be mentioned that sulfonamides can trigger environmental concerns, such as accumulation in fish and birds, and they can lead, even at trace levels, to the development of antimicrobial resistance [6,7]. Therefore, considering the incapacity of conventional processes in sewage treatment plants to completely remove sulfonamides, plus the negative environmental impacts, research on alternative processes to deal with sulfonamides, such as SMX and SAM, in aqueous media must be performed.

To address the challenge of dealing with organic pollutants in water, diverse treatment technologies have been explored, including biological methods (using microorganisms, algae, or enzymes), physical techniques (e.g., filtration, reverse osmosis, and adsorption) and chemical processes (e.g., Fenton reactions and advanced oxidation processes which are based on the generation of strong oxidizing agents) [14]. In fact, photocatalytic advanced oxidation processes involving heterogeneous catalysts (e.g., hybrid nanomaterials or even materials obtained from solid waste) have been explored to see if they degrade antibiotics in water samples [15,16]. However, this kind of process has the main drawback of the recovery of the photocatalyst. Then, other alternative advanced oxidation processes should be evaluated. For instance, physical–chemical processes that use ultrasound (mechanical waves) or electrochemistry (electron movements) are promising alternatives to degrade active pharmaceutical compounds in water [6,17]. High-frequency ultrasound (>200 kHz) induces acoustic cavitation (represented by “*]]]”* in Equation 1). This promotes the formation, growth, and collapse of bubbles, leading to oxygen and water vapor cleavages producing hydroxyl radicals (HO**•**, Equation (1)), which are strong oxidizing species able to degrade antibiotics (S, see Equation (2)) [17]. In the case of the electrochemical, the pollutant (S) degradation can occur through the oxidation on an anode surface via electron releasing (Equation (3)) or by the action of oxidizing species (M_ox_) electrogenerated from an aqueous medium (M, e.g., supporting electrolyte; Equations (4) and (5)) [18].

H_2_O + *]]]* → HO**•** + H**•** (1)

S + HO**•** → degradation products (2)

Anode + S → Products + ne-(3)

Anode + M → M_ox_ (4)

M_ox_ + S → Products (5)

Some previous works have reported the use of ultrasound, mainly low-frequency waves (<200 kHz) in combination with ozone, hydrogen peroxide, or electro-oxidation for degrading SMX [19,20,21,22]. However, to the best of our knowledge, systematic studies about the application of mid-high-frequency ultrasound (200–500 kHz, which is the region of the highest sonogeneration of radicals [23,24]) and comparisons with other processes have been little studied. Only two papers have considered the SMX treatment by sonochemistry at high frequencies (580 and 1000 kHz), but these works were primarily aimed at degrading the antibiotic sono-catalytically [25,26]. Furthermore, comparisons of the treatment of sulfonamide degradation are also scarce.

Electrochemical processes using metal oxides of Ir and/or Ru as electrodes have also been tested for degrading SMX [6], but the comparison of such processes with mid-high-frequency ultrasound, in terms of their intrinsic capabilities (e.g., action routes, nature of the sulfonamide, and acceleration or inhibition of degradation according to the matrix and target pollutant) has not been reported yet. Moreover, low-cost computational calculations (such as atomic charge analyses) to identify reactive moieties on sulfonamides toward degrading species are not usually utilized in the above-presented works. Additionally, the comparison of cyclic sulfonamide (SMX) degradation with another noncyclic sulfonamide (SAM) has not been studied in the mentioned literature [6,19,20]. Thereby, in this research, such missing aspects were considered. Furthermore, theoretical calculations about the biological activity of the possible transformation products coming from the treatment of the target sulfonamides are studied herein.

## 2. Results and Discussion

### 2.1. Treatment by Ultrasound

#### 2.1.1. Effect of Ultrasound Power on the Sonochemical System

**Figure 1:**
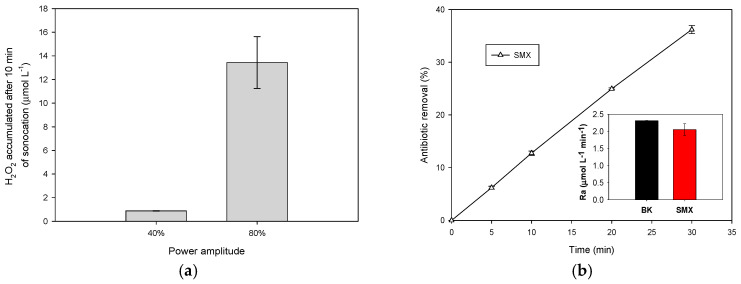
Sonochemical treatment of SMX. (**a**) Effect of ultrasound power amplitude on the sonogeneration of H_2_O_2_. (**b**) Determination of the main degradation route (BK: blank, distilled water in the absence of sulfonamides).

Before starting the degradation of the target sulfonamides, the effect of the ultrasound power amplitude on the radicals’ sonogeneration by the ultrasound system was considered. It should be indicated that in the ultrasonic process, in the absence of pollutants, hydroxyl radicals combine to produce hydrogen peroxide (Equation (6)), and its accumulation is considered an indicator of sonochemical activity [24,27]. Then, to obtain a proper operative value for the sonochemical power amplitude that generates a high amount of hydroxyl radicals, the accumulation of H_2_O_2_ at 40% and 80% ultrasound power amplitude was measured at 10 min of sonication (Figure 1a).

HO**•** + HO**•** → H_2_O_2_ (6)

As presented in Figure 1a, at 80% ultrasound power amplitude, the H_2_O_2_ accumulation (i.e., the production of HO**•**) was higher. Indeed, the H_2_O_2_ accumulation at 80% is ~15 times higher than when 40% was applied. This is explained by a higher amount of cavitation events and collapsing bubbles, as the power increases, resulting in higher radical and H_2_O_2_ production [28]. Hence, 80% ultrasound power amplitude was chosen to evaluate the sonodegradation of the sulfonamides.

#### 2.1.2. Routes Involved in the Sonodegradation Process

The degrading action of the ultrasound on SMX in distilled water was initially tested. Figure 1b shows the evolution of the antibiotic removal for 30 min of treatment. This process achieved ~37% of the pollutant degradation. It can be mentioned that ultrasound as a degradation system comprises three action zones of the cavitation bubbles: a. the inner part of the bubble; b. the bubble–solution interface; and c. the solution bulk. Depending on the nature of the pollutant, the degradation occurs in a specific zone. Volatile compounds are degraded by pyrolysis in zone a, hydrophobic substances are eliminated in the interface (zone b) by attacks of HO**•**, and in zone c, hydrophilic compounds are degraded by some hydroxyl radicals ejected during the collapse of the cavitation bubble.

The rates of hydrogen peroxide accumulation values (Ra) during the SMX treatment were determined and compared with the same parameter in the absence of the antibiotic. As seen in the inset in Figure 1b, the Ra value in the presence of SMX is lower than in its absence (BK), indicating that the sonodegradation of SMX is promoted by hydroxyl radicals [17,24]. Additionally, as SMX is a non-volatile compound, degradation via pyrolysis is discarded. According to LogP (the logarithm of the octanol/water partition coefficient), which is an indicator of hydrophobicity, a low LogP value denotes a high hydrophilic substance [29]. SMX is a moderately hydrophilic compound, as this pharmaceutical has a LogP = 0.48/0.89 [30,31]. Due to the moderate hydrophilic nature of SMX, it is mainly degraded in the solution bulk by attacks of hydroxyl radicals [30,31].

### 2.2. Degradation by the Electrochemical Treatment

#### 2.2.1. Effect of the Current on the Electrogeneration of Oxidizing Species

The capability of the electrochemical system to electrogenerate oxidizing species was evaluated at two different current intensities (12 and 40 mA). In a solution containing NaCl, the Ti/IrO_2_ anode oxidizes chloride ions toward chlorine molecules (Equation (6)) [6,32]. Subsequently, the chlorine molecules are converted into hypochlorous acid (or hypochlorite anion) and hydrochloric acid (Equations (7)–(9)) [33]. HClO and ClO^−^ are oxidizing species capable of attacking organic pollutants [34]. At the experimental pH (which started at 6.5 and during the process decreased to 4.5), HClO is the predominant chlorine species in the aqueous solution.

Ti/IrO_2(anode)_ + 2Cl^−^ → Cl_2_ + 2e^−^ (7)

Cl_2_ + H_2_O → HClO + HCl (8)

HClO + H_2_O → H_3_O^+^ + ClO^−^, pKa: 7.5 (9)

**Figure 2:**
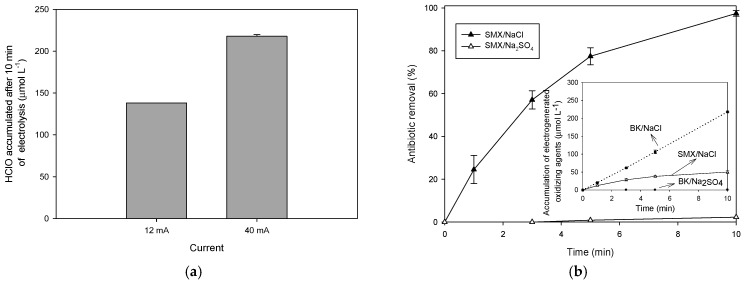
Electrochemical treatment of SMX. (**a**) Effect of the current intensity on the electrogeneration of oxidizing/degrading species (HClO). (**b**) Sulfamethoxazole degradation by the electrochemical process in the presence of two supporting electrolytes (NaCl or Na_2_SO_4_); inset: accumulation of oxidizing species in distilled water in the absence (BK) and presence of the antibiotic (SMX).

Figure 2a shows the HClO accumulation after 10 min of electrolysis. We can observe that as the current intensity changed from 12 to 40 mA, the formation of HClO increased, because more electrons can be removed from the chloride ion to promote the chlorinated oxidative species (Equations (7)–(9)) [35]. However, the accumulation of these species was not completely linear because the increase in the current density can promote parasitic reactions such as the oxygen evolution from water oxidation (Equation (10) [35]) in addition to the chlorine formation (Equation (7)). Then, despite the parasitic reaction of oxygen evolution, the formation of the HClO was higher at 40 mA; thus, this condition was selected to perform the SMX treatment. Also, it is important to mention that at very high current intensities (>100 mA), highly oxidized chlorine species (e.g., chlorate and perchlorate anions) could be formed, and such strongly oxidized chlorine species could act as degrading species [36], but under our experimental conditions, this aspect is discarded.

Ti/IrO_2(anode)_ + 2H_2_O → O_2_ + 4H^+^ + 4e^−^ (10)

#### 2.2.2. Routes Involved the Electrodegradation Process

SMX was treated by the electrochemical system utilizing NaCl as a supporting electrolyte and compared to the system using Na_2_SO_4_. The first supporting electrolyte allows us to identify the degrading role of electrogenerated oxidizing species. Meanwhile, the second one reveals the contribution of the direct oxidation of pollutants on the anode surface [32]. As Figure 2b shows, when NaCl is the supporting electrolyte, ~98% of SMX was degraded after 10 min of electrolysis. In contrast, with Na_2_SO_4_ as the conductive medium, less than 5% of the sulfonamide antibiotic was removed over the same treatment time. As mentioned above, the electrochemical system in the presence of NaCl generates HClO, which is an oxidizing species capable of attacking and degrading SMX [34,37].

Besides the degradation of the antibiotic, the evolution of oxidizing species (i.e., HClO/ClO^−^) in distilled water, utilizing NaCl as a supporting electrolyte in the presence and absence of SMX, was followed (inset in Figure 2b). The accumulation in the antibiotic’s presence was lower than in its absence, and this confirmed the degrading role of HClO/ClO^−^. Interestingly, as observed in the inset in Figure 2b, once the antibiotic is practically eliminated (i.e., after 10 min of treatment), the accumulation of oxidants increases significantly, which suggests a poor degradation of SMX byproducts by HClO. Moreover, in the presence of sodium sulfate, there was no formation of oxidizing species. Thereby, the little degradation observed when Na_2_SO_4_ was used as the supporting electrolyte (lower than 5%) denotes a small contribution of the SMX oxidation on the anode surface [32].

**Figure 3:**
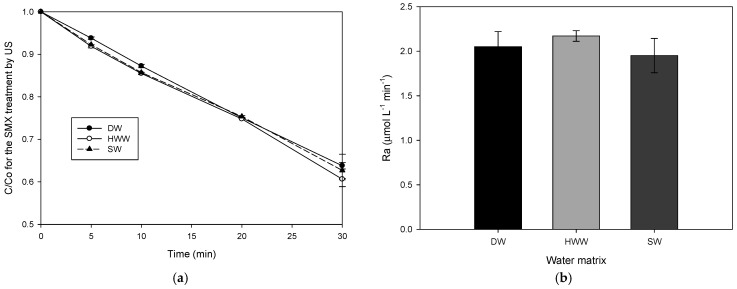
Comparison of the SMX degradation in DW (distilled water), HWW, and SW by sonochemistry. (**a**) Evolution of SMX in the diverse matrices. (**b**) H_2_O_2_ accumulation rate in DW, HWW, and SW in the sulfonamide presence.

After evidencing the ability of the ultrasonic and electrochemical systems to degrade SMX in distilled water (DW), the elimination of this sulfonamide in two synthetic complex matrices (i.e., hospital wastewater—HWW and seawater—SW, Table 1) was assessed. As mentioned in the Introduction section, sulfonamide antibiotics are frequently found in these kinds of aqueous matrices. Figure 3a compares the evolution of the antibiotic in DW, SW, and HWW under sonochemical treatment. It was found that the sonodegradation of SMX in the hospital wastewater and seawater was very similar to that obtained in distilled water (~35% after 30 min of treatment), suggesting that the other components of the complex matrices had no effects on the action of the ultrasound system. In addition to the SMX evolution, the accumulation rate of H_2_O_2_ in the antibiotic’s presence was determined for the three matrices (Figure 3b), also showing very close values, which confirms that there is negligible competition of matrix components with the sonogenerated radicals.

**Figure 4:**
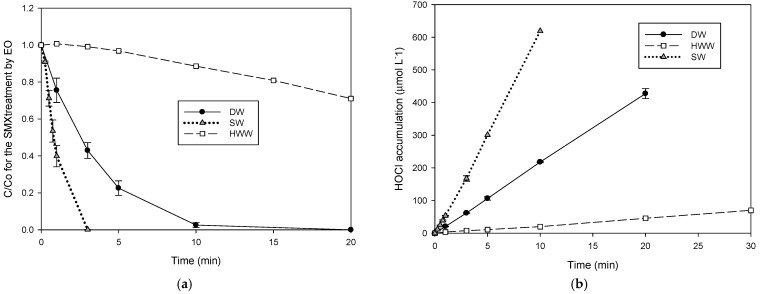
Comparison of the SMX degradation in DW, HWW, and SW by electrochemistry. (**a**) Evolution of the sulfonamide; (**b**) HClO accumulation in DW, HWW, and SW in the sulfonamide absence.

In turn, Figure 4a presents the SMX degradation in HWW and SW by the electrochemical process. The SMX degradation in SWW was accelerated compared to the antibiotic elimination in DW. In contrast, the treatment in the hospital wastewater revealed that this matrix inhibited sulfonamide degradation, denoting the competition of substances in the complex matrix by the electrogenerated degrading species [32]. Figure 4b depicts the HClO accumulation in the different matrices in the absence of SMX, evidencing that the SW matrix led to a very high production of hypochlorous acid, while in HWW, the HClO accumulation is lower than in DW.

The explanation for the low-interference matrix effects in the ultrasound system relies on the hydrophilicity of the hospital wastewater or seawater components. It can be noted that the other substances apart from SMX are hydrophilic (Log *p* values < 0, Table 1). Thus, according to their low Log *p* values, these substances are mainly placed in the solution bulk, whereas SMX (with a Log *p* value higher than the other matrix components) is closer to the cavitation bubble. Consequently, the matrix components in the hospital wastewater did not interfere with the antibiotic degradation (Figure 3a) [17,28], leading to a selective degradation of SMX by ultrasound, and for the same reason, the H_2_O_2_ accumulations in these matrices were similar to that obtained for DW (Figure 3b).

On the contrary to the sonochemical process, in the electrochemical system, the antibiotic elimination in seawater was strongly accelerated, and the SMX degradation in the hospital wastewater was partially inhibited. The acceleration of the sulfonamide degradation is associated with the fact that SW is a chloride anion-rich matrix (~550,000 µmol L^−1^ of Cl^−^); this anion is a precursor for the electrogeneration of HClO, and it also favors electrical conductivity (which is also a key parameter for electrochemical systems and for the characterization of diverse water samples [38]). Consequently, a high amount of HClO is quickly electrogenerated (as evidenced in the control experiment for the HClO accumulation in SW matrix, Figure 4b). Meanwhile, the inhibition of the pharmaceutical elimination in HWW can be understood through a strong interaction of electrogenerated HClO [34] with the matrix components. For instance, HWW has high concentrations of urea and ammonium, and these components can easily react with active chlorine species (Equations (11) and (12) [39]), thus slowing SMX degradation. Indeed, the interaction of urea and ammonium with the electrogenerated HOCl was experimentally confirmed by a lower active chlorine species accumulation in HWW compared to DW (Figure 4b).

(11) $${\left(NH_{2}\right)}_{2}CO+3HOCl\to CO_{2}+N_{2}+3Cl^{-}+2H_{2}O+3H^{+}$$

(12) $$2NH_{4}^{+}+3HOCl\to N_{2}+3H_{2}O+5H^{+}+3Cl^{-}$$

### 2.3. Comparison of the Two Processes for Dealing with the Representative Sulfonamides

#### 2.3.1. Nature of the Chemical Structure of the Pollutant

**Figure 5:**
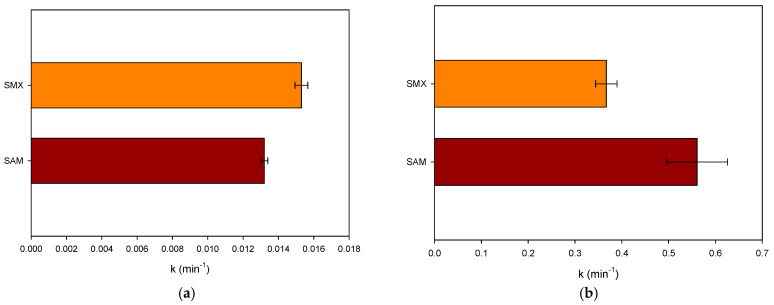
Nature of the sulfonamide effect. (**a**) Comparison of the pseudo-first-order rate constant for SMX and SAM treated by sonochemistry in distilled water. (**b**) Comparison of the pseudo-first-order rate constant for SMX and SAM treated by electrochemistry in distilled water.

To evaluate the role of the antibiotic structure, a representative noncyclic sulfonamide (sulfacetamide—SAM) was considered and treated in distilled water by the two processes individually. Moreover, the sonodegradation of SMX was compared with the treatment of SAM. Figure 5a presents the comparison of the pseudo-first-order constants for the degradation of SMX and SAM by ultrasonic action. We can note that SMX is faster (k_SMX_: 0.0153 min^−1^, R^2^: 0.998) than SAM (k_SAM_: 0.0132 min^−1^, R^2^: 0.999). Both antibiotics have the same sulfonamide nucleus, but the degradation of SAM is slower than the elimination of SMX. SAM has an acetamide group (while SMX has an isoxazole moiety), which makes SAM more hydrophilic (LogP = −0.96/−0.60 [30,31]). Hence, SMX is degraded faster than SAM, thus confirming that SMX is more easily degraded by ultrasound than the other, more hydrophilic sulfonamide.

When the electrochemical treatment of SMX (k: 0.3670 min^−1^, R^2^: 0.989) was compared with the elimination of the other sulfonamide antibiotic (SAM, k: 0.5610 min^−1^, R^2^: 0.974), it can be noted that the electrochemical degradation of the former is slower than the latter (Figure 5b). Some works about the degradation of sulfonamides have shown that the main chlorine species attack can be expected at the aniline group, and in the case of SMX, the heterocycle (i.e., isoxazolyl moiety) has low reactivity toward chlorine [40]. Thus, due to the higher pseudo-first-rate constant observed for SAM, the acetamide group could be expected as another reaction site for this molecule. This result indicates that other compounds having structures with moieties that are very reactive toward the electrogenerated chlorine species will be degraded faster than SMX by the considered electrochemical process.

From the above results, we can say that degradation of the two sulfonamides by the sonochemical process is governed by hydrophobicity and the attacks of the HO**•**. Meanwhile, in the electrochemical treatment, the reactivity of functional groups toward the electrogenerated HClO is a determinant for the degradation of the target sulfonamides (Table 2).

#### 2.3.2. Theoretical Analyses of the Reactive Moieties on the Target Sulfonamides

**Figure 6:**
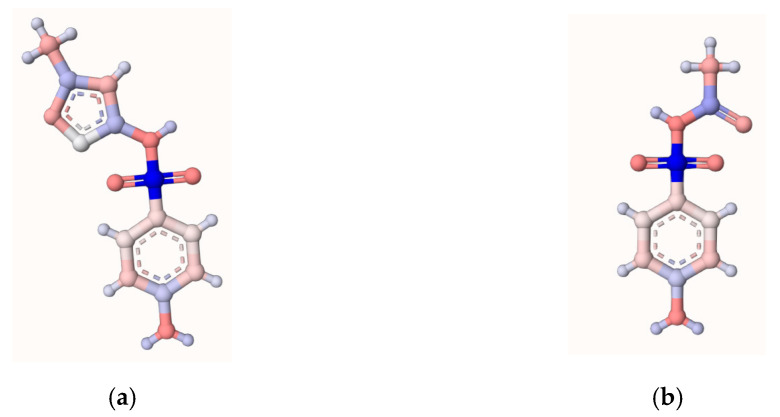
Theoretical analysis of atomic charge for the target sulfonamides (**a**) SMX and (**b**) SAM. Atoms in red are electron-richest moieties, and they are more susceptible to attack by HO**•** or HClO.

To study the transformations of SMX, initially, the electron-rich regions on SMX that are more susceptible to transformations by attacks of oxidizing species were determined using computational analyses [41,42,43]. Thus, calculations using the atomic charge calculator tool (ACC, which is a free online tool) were performed (the AtomicChargeCalculator II freeware was used [44]). The computational analyses showed that the central S-N bond, the N and aromatic ring in the aniline moiety, plus the C=C bond and methyl groups in the isoxazole structure were the moieties of SMX that had more electron density (Figure 6a). For SAM (Figure 6b), the calculation of the atomic charges revealed that the central sulfonamide S-N bond, the aromatic ring, and the aniline group were also the electron-richest moieties. Additionally, the methyl group of the acetamide portion can be susceptible to attacks by electron-deficient species such as HO**•** or HClO.

**Figure 7:**
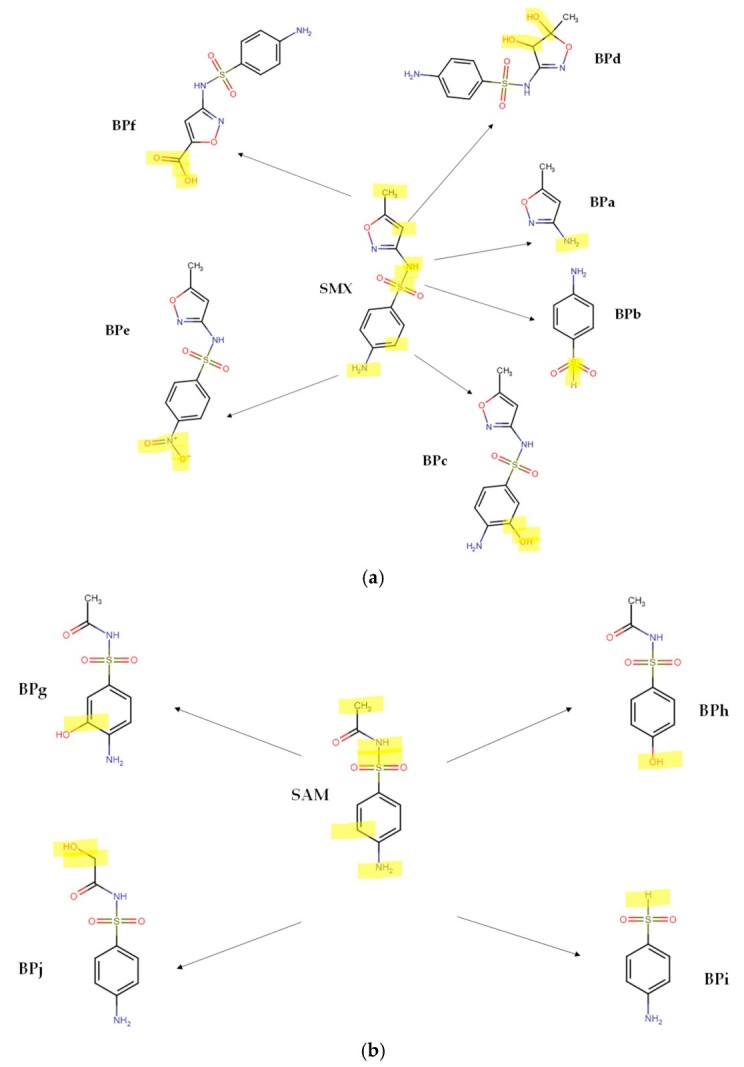
Possible byproducts (BPs) from the attacks of HO**•** on the target sulfonamides. (**a**) SMX (figure based on the references [19,45]); (**b**) SAM (figure based on the reference [46]). Note: the moieties highlighted in yellow color are those that have being transformed by the process.

**Figure 8:**
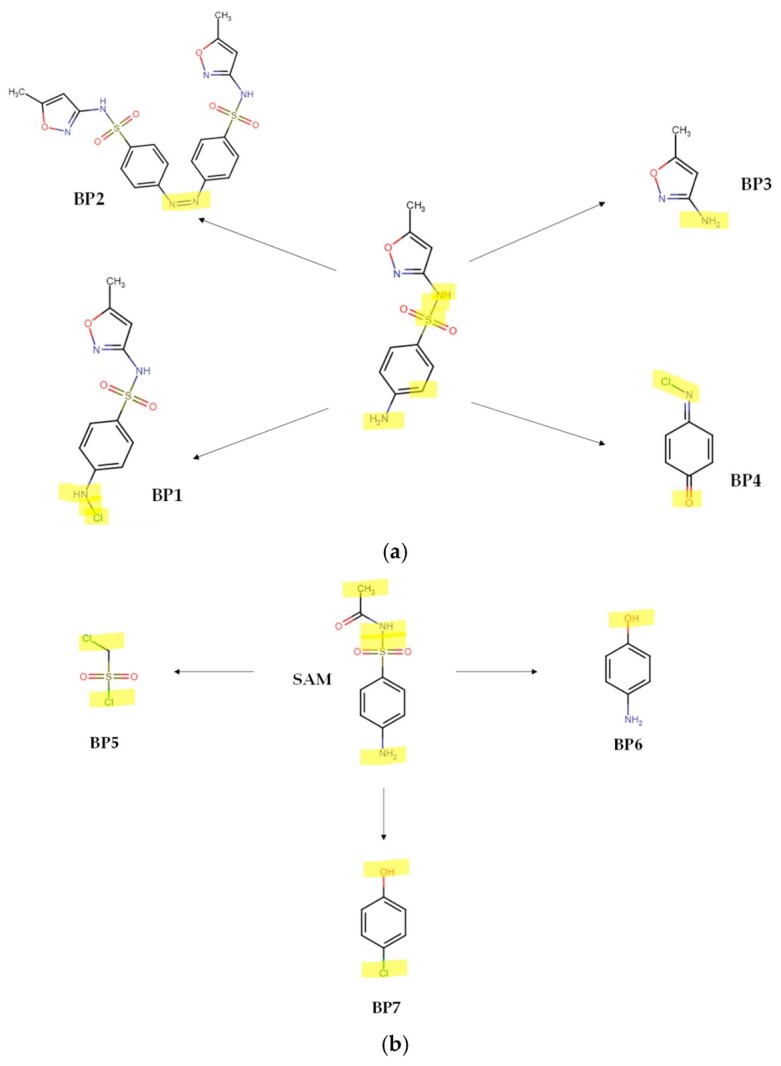
Typical byproducts (BPs) from the attacks of HClO on the target sulfonamides. (**a**) SMX (figure based on the references [37,40]); (**b**) SAM (figure based on the reference [47]). Note: the moieties highlighted in yellow color are those being transformed by the process.

The electron-rich regions of the sulfonamide antibiotics are attacked and transformed by the sonogenerated HO**•** or the electrogenerated HClO. Our theoretical results are consistent with the previous literature that reports the experimental formation of primary intermediates (BP) from the sonodegradation of SMX (Figure 7a), which comes from the S-N cleavage (BPa and BPb), hydroxylation of the benzene ring (BPc), hydroxylation of the C=C bond in the isoxazole ring (BPd), oxidation of the amine group on the aniline structure (BPe), and oxidation of the methyl group at the isoxazole ring (BPf) [19,45]. The literature also reports that active chlorine species, such as HClO, are able to react with the aniline moiety of SMX, inducing a chlorination (BP1) or dimerization (BP2) of the pollutant [40]. Furthermore, the rupture of the SMX sulfonamide moiety to yield 3-amino-5-methylisoxazole (BP3, which is the same as BPa) and *N*-chloro-*p*-benzoquinone-imine (BP4) has been informed (Figure 8a) [37].

According to previous works on the treatment of sulfonamides by processes based on radical species, the attacks by HO**•** on SAM could firstly induce the hydroxylation of its aromatic ring (BPg, Figure 7b), substitution of the -NH_2_ group by the -OH group to form BPh, in addition to the cleavage of the S-N bond (BPi), and the oxidation of the methyl group (BPj) [46]. Meanwhile, the reaction of SAM with HClO (Figure 8b) initially leads to the breakdown of the C–S bond between the sulfonyl group and benzene ring and the S-N bond between sulfonyl and acylamino groups with the subsequent generation of chloro-methane-sulfonyl chloride (BP5), 4-hydroxyaniline (BP6), and 4-chlorophenol (BP7) [47].

At this point, it is relevant to recognize that the initial focus of our work was to show the usefulness of the ACC theoretical tool, connecting it with the possible primary products based on the available literature. However, for a deeper discussion, future analytical work employing liquid chromatography coupled to mass spectrometry (LC-MS) must be performed to confirm the actual byproducts formed (and propose the degradation mechanisms) in our sonochemical and electrochemical systems.

**Figure 9:**
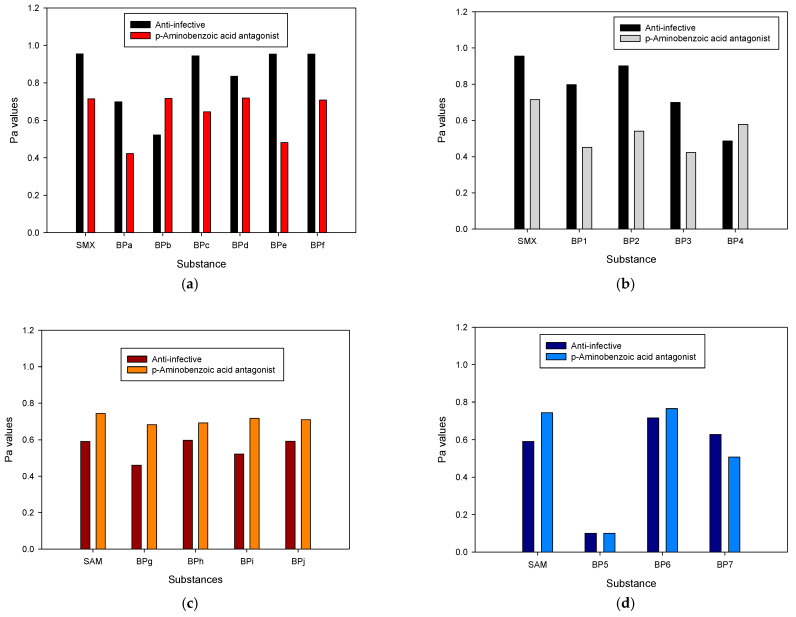
Predictions of antimicrobial activity for the sulfonamides and their possible byproducts. For SMX: (**a**) sonochemical process, (**b**) electrochemical process. For SAM: (**c**) sonochemical process, (**d**) electrochemical process.

To study the possible risks associated with the primary byproducts (e.g., promotion of antimicrobial resistance or toxic effects), biological activities such as anti-infective capability and their action as a p-aminobenzoic acid antagonist were considered. Thereby, for the considered byproducts coming from the hydroxyl radical attacks on SMX, the probabilities of being active (Pa values) were estimated theoretically. As shown in Figure 9a, BPa, BPb, and BPd had Pa values slightly lower than SMX for anti-infective capability, while the other byproducts showed Pa values similar to the parent antibiotic. At the same time, some byproducts (BPb, BPc, and BPe) exhibited slightly lower Pa values than SMX for the p-aminobenzoic acid antagonist biological activity, but Pa for the other ones was close to that of the parent sulfonamide. These results could be explained by considering that most of these primary byproducts retained the antibiotic nucleus (i.e., the central sulfonamide moiety).

Figure 9b shows the probabilities of being active (Pa values) for the possible byproducts coming from the interaction of SMX with HClO. The four considered byproducts exhibited Pa values lower than the parent sulfonamide. However, the Pa values for the byproducts were above 0.4, which denotes that they are still very active. On the other hand, the predictive analysis for the biological activities of SAM and its possible byproducts was also considered (Figure 9c,d). BPg and BPi exhibited lower Pa values for the anti-infective activity, while the other primary byproducts presented Pa values close to the parent sulfonamide (Figure 9c), thus suggesting that only the modification of the hydroxylation of the aromatic ring of SAM and the cleavage of the S-N bond would lead to a relevant decrease in the antimicrobial activity. Moreover, all the primary byproducts from the radical-based process showed similar Pa values to SAM for the p-aminobenzoic acid antagonist activity (Figure 9c). Our theoretical analyses fit well with findings by other authors, which report that byproducts maintaining the sulfonamide structure can induce toxic effects [45,48].

In contrast to the possible primary byproducts from the radical-based process (Figure 7b), those from the electrochemical process did not retain the sulfonamide structural nucleus (Figure 8b), and only BP5 presented substantially lower Pa values than SAM (Figure 9d). Furthermore, from the theoretical results in Figure 9d, it can be observed that other small molecules such as 4-hydroxyaniline (BP6) and 4-chlorophenol (BP7) could also be active against bacteria. Indeed, 4-chlorophenol (4-CP) is a well-known toxic and broad-spectrum antiseptic, while 4-hydroxyaniline (also called 4-aminophenol or 4-AP) has potential in creating antimicrobial agents. 4-CP is highly effective against bacteria due to its ability to disrupt membranes, whereas 4-hydroxyaniline derivatives show selective antibacterial activity [49,50].

Due to the potential biological activities of some byproducts, alternatives to degrade risky primary intermediates should be developed. A strategy could be the addition of iron ions to get a sono-Fenton, which enhances the production of radicals from the sonogenerated H_2_O_2_ in the solution bulk [24], leading to the degradation of primary byproducts toward simpler biocompatible substances (e.g., short-chain carboxylic acids) [51]. Likewise, the addition of iron to the electrochemical process could produce radical species (via HClO cleavage) that induce strong structural changes [52] and then modify the biological activity. Finally, it is important to note that the prediction of the biological activities for the possible byproducts from the sulfonamides’ degradation is a first approach; thus, it is suggested to carry out further experimental research about this topic to verify the theoretical predictions.

## 3. Materials and Methods

### 3.1. Reagents

Sulfamethoxazole was provided by Sigma (St Louis, MO, USA). Sulfacetamide was acquired from Corpaul (Guarne, Colombia). Sodium bicarbonate, sodium chloride, sodium sulfate, potassium dihydrogen phosphate, calcium chloride dihydrate, magnesium chloride hexahydrate, potassium chloride, potassium bromide, ammonium chloride, urea, and acetonitrile were purchased from Merck (Darmstadt, Germany). Formic acid was provided by Carlo-Erba (Val de Reuil, France). All chemicals were used as received. For the experiments, the solutions were prepared using distilled water. The simulated hospital wastewater (HWW) and seawater (SW) were prepared as detailed in Table 1, and the pH was 6.5 (according to references [32,53]).

### 3.2. Reaction Systems

The samples (300 mL, at 40 µmol L^−1^ of the antibiotic and pH 6.5) were treated using an ultrasound reactor (Meinhardt Ultrasonics^®^, Leipzig, Germany) operated at 375 kHz of frequency, at 80% power amplitude; the reactor delivers 88 W L^−1^ of actual ultrasonic power density (measured by calorimetric method [54]). The reactor temperature was controlled at 20 °C using a Huber Minichiller. These experimental conditions were selected based on previous works from our research team [17,24,28]. In the electrochemical system, an undivided cell of 150 mL operated under constant stirring conditions was utilized. A Ti/IrO_2_ rectangular plate of 8 cm^2^ (anode) and a zirconium spiral of 10 cm^2^ (cathode) were used for the experiments. It can be mentioned that despite electrodes based on noble metals such as iridium being relatively expensive, they can be less costly than the boron-doped diamond electrodes (BDD, which are also used in oxidative processes for degrading pollutant in water); additionally, the Ti/IrO_2_ electrodes can have the highest dimensional stability and a stronger catalytic activity than BDD toward the electrogeneration of active chlorine species such as HClO. In this work, the current intensity was variable, and supporting electrolyte was used at 0.05 mol L^−1^. These experimental conditions were selected based on previous work from our research team [32].

### 3.3. Methods

#### 3.3.1. Experimental Analyses

Quantitative analysis of antibiotics was carried out using a UHPLC Thermo Scientific Dionex UltiMate 3000 instrument (Thermo Fisher Scientific, Bartlesville, OK, USA) equipped with an Acclaim^TM^ 120 RP C18 column (5 µm, 4.6 × 150 mm) and a diode array detector (Table 3). The injection volume was 20 µL, and mixtures of acetonitrile (ACN)/formic acid (FA, at 10 mmol L^−1^, pH 3.0) were used as mobile phase.

The degradations of the antibiotics by ultrasound and electrochemistry follow pseudo-first-order kinetics [17,55]. Then, the respective rate constants (k) were determined as the slope of the plot of Ln (C/Co) vs. time, and the R^2^ value for the linear regression was used as the goodness-of-fit criterion. Such rate constants were calculated for the degradation of the target pollutants in distilled water (DW) and HWW. On the other hand, the accumulations of the sonogenerated hydrogen peroxide and the initial rate of H_2_O_2_ accumulation (Ra) were determined as described in the reference [24]. The measurement of the electrogenerated oxidizing species (e.g., HClO) was made according to the iodometric/spectrophotometric method described in the reference [35].

#### 3.3.2. Theoretical Analyses

The regions on the antibiotics that are more susceptible to electrophilic attacks by degrading agents were identified using theoretical analyses of atomic charge (ACC, which provides an initial approach to the electron density on the target sulfonamide). The ACC was performed through the AtomicChargeCalculator II freeware by uploading the structure of the antibiotic in an SDF format (ACC II was used (https://acc2.ncbr.muni.cz/ (accessed on 14 December 2024)). The AtomicChargeCalculator II freeware is based on the implementation of the Electronegativity Equalization Method (EEM); the atomic charges are calculated from the 3D structure of the target molecule (in the SDF format). It must be noted that this is an empirical non-quantum mechanics method (more details about the freeware can be obtained from the references [56,57]).

In addition to ACC calculations, predictions of the antimicrobial biological activity of the target sulfonamide and the primary degradation products (which were taken from the literature [19,37,40,45]) were calculated using the PASS freeware. It must be mentioned that PASS possesses structure–activity relationships (SARs) on a conceptual basis. The chemical structure is uploaded to the PASS software (Way2Drug.com©, Version 2.0) in the SMILE format. Subsequently, the values of the probability of biological activities (Pa) for the tested compounds are determined [44]. It is important to mention that the prediction is based on the SAR analysis using more than 250,000 biologically active compounds, including drugs, drug candidates, leads, and toxic substances [58]. The prediction average accuracy involves the leave-one-out cross-validation procedure (more details are provided in the reference [59]).

## 4. Conclusions

The target sulfonamides were effectively degraded in water samples by sonochemistry and electrochemistry, where the main degrading role in ultrasound was played by HO**•** coming from the acoustic cavitation; meanwhile, the HClO from the oxidation of chloride anions at the anode was the predominant elimination route in the electrochemical process. The sulfonamide degradation in distilled water fits well with pseudo-first-order kinetics, having rate constants of 0.0153 and 0.0132 min^−1^ for the sonochemical treatment of SMX and SAM, respectively. Meanwhile, the electrochemical degradation had rate constants of 0.3670 and 0.5610 min^−1^ for SMX and SAM, respectively. The sonodegradation of SMX was more efficient than that of SAM due to the higher hydrophobicity of sulfamethoxazole. Meanwhile, the electrochemical degradation of SMX was slower than that of SAM because of the lower reactivity of the isoxazolyl ring on SMX compared to the acetamide moiety on SAM. Thanks to the highly hydrophilic nature of the HWW and SW components, SMX was selectively sonodegraded in such an aqueous matrix, whereas the electrochemical process performance was strongly improved by the SW matrix (due to a very high production of HClO), but it was affected in HWW owing to the competing reactions of the HWW components with the electrogenerated HClO. Moreover, the atomic charge analyses of the moieties most susceptible to the attacks by the sonogenerated hydroxyl radical agreed well with experimental results reported in the literature. Additionally, it must be mentioned that most of the plausible primary intermediates retain the central sulfonamide moiety; however, their probability of being active against bacteria is slightly lower than that of the parent sulfonamide pharmaceutical. Therefore, it is recommended for further work to perform a sono-Fenton or electro-Fenton process for increasing the production of radical species to favor strong transformations of the target pollutants.

## Figures and Tables

**Table 1:** Composition of the synthetic hospital wastewater (HWW) and seawater (SW).

HWW							
Substance	Urea	NaCl	KCl	NH_4_Cl	Na_2_SO_4_	KH_2_PO_4_	CaCl_2_-2H_2_O
Concentration (µmol L^−1^)	21,000	51,300	1340	940	710	370	340
Log *p*	−2.59	−0.46	0.20	−0.21	−0.84	−3.96	−0.57
**SW**							
Substance	NaHCO_3_ *	NaCl	KCl	KBr *	Na_2_SO_4_	MgCl_2_-6H_2_O *	CaCl_2_-2H_2_O
Concentration (µmol L^−1^)	2393	500,000	9323	849	28,795	54,594	10,448

* Log *p* values < 0.

**Table 2:** Comparison of the two sulfonamides’ degradation by sonochemistry and electrochemistry.

Process	Sonochemistry	Electrochemistry
Predominant degradation route	HO**•**	HClO
Indicator parameter of pollutant degradation	Hydrophobicity of the sulfonamide	Reactive sites on the sulfonamide
Favored pollutant	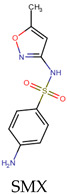	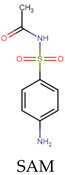

**Table 3:** Chromatographic conditions for antibiotics analysis.

Antibiotic	Flow (mL min^−1^)	Mobile Phase (%ACN/%FA)	Detection Wavelength (nm)
SMX	0.6	40/60	270
SAM	0.6	40/60	257

## Data Availability

Data will be available on request to the authors.

## Abbreviations

The following abbreviations are used in this manuscript:

SMX	Sulfamethoxazole
SAM	Sulfacetamide
DW	Distilled water
HWW	Synthetic hospital wastewater
SW	Synthetic seawater
k	Pseudo-first-order rate constant
BK	Blank
BP	Byproduct
Ra	Rate of accumulation
Log P	Logarithm of the octanol/water partition coefficient
ACC	Atomic charge calculator
BDD	Boron-doped diamond
